# A Single Dose of Amoxicillin and Dexamethasone for Prevention of Postoperative Complications in Third Molar Surgery: A Randomized, Double-Blind, Placebo Controlled Clinical Trial

**DOI:** 10.4021/jocmr1160w

**Published:** 2013-01-11

**Authors:** Marcelo Carlos Bortoluzzi, Diogo Lenzi Capella, Tharzon Barbieri, Micheli Pagliarini, Talita Cavalieri, Rafael Manfro

**Affiliations:** a School of Dentistry, Health Bioscience Postgraduate Program, Tissue Aspects for Health Prognosis and Intervention Laboratory (LAPROG), Oeste de Santa Catarina University (UNOESC), Brazil; b School of Dentistry, Oeste de Santa Catarina University (UNOESC), Brazil; c Oeste de Santa Catarina University (UNOESC), Brazil

**Keywords:** Third molar surgery, Antibiotic prophylaxis, Amoxicillin, Dexamethasone

## Abstract

**Background:**

The aim of this study was to assess the efficacy of a single prophylactic dose of amoxicillin and/or dexamethasone in preventing postoperative complications (PC) after a surgical removal of a single mandibular third molar (M3).

**Methods:**

This study is a randomized, placebo controlled clinical trial. Four groups were included: Group 1 (G1) included a prophylactic dose of 2 g of amoxicillin and 8 mg of dexamethasone; Group 2 (G2) included a prophylactic dose of 2 g of amoxicillin and 8 mg of placebo; Group 3 (G3) included a prophylactic dose of 8 mg of dexamethasone and 2 g of placebo and; Group 4 (G4) placebo.

**Results:**

Fifty patients were included. It was observed one case of alveolar infection (2%) and two of alveolar osteitis (4%) resulting in three PC (6%). No statistical differences were observed between therapeutic groups for development of PC, trismus, pain and edema. The use of antibiotics showed an absolute risk reduction (ARR) for PC development of 3.52% and the number needed to treat (NNT) was 29.

**Conclusion:**

Prophylactic antibiotics and corticoid in a single dose regimen did not bring any benefit on M3 surgeries.

## Introduction

The incidence of postoperative complications (PC) following mandibular third molar (M3) extraction, such as alveolar osteitis (AO) or alveolar infection (AI), have been reported in different frequencies and extents, ranging from mild discomfort after the operation to major complications including death [1-12]. Apart from surgical complications, removal of third molars is also frequently associated with considerable postoperative discomfort such as pain trismus and edema [4, 5, 13, 14].

Due to high index of PC and discomfort after M3 surgeries, many dentists routinely prescribe antibiotics and corticosteroids based on generalized and nonspecific recommendations, however the routine use of antibiotics for the removal of asymptomatic third molars is controversial. While there is some evidence that antibiotics can reduce the incidence of PC [1, 3], there is equally evidence to the contrary [10-12, 15, 16]. Facing the controversy some authors also ponder that antibiotics may be efficacious in reducing the incidence of PC following third molar extraction but should not be prescribed in all cases [1]. Moreover different methodological approach has been described, like Arteagoitia et al [1] who prescribed prophylactically amoxicillin/clavulanic acid 500/125 mg oral 3 times a day for 4 days after the intervention and, Ataoglu et al [16] whom used two different regimens, 1 g with clavulanic acid orally twice daily starting directly after operation for 5 days and a second group had the same regimen but starting 5 days before operation. In many studies antibiotics are prescribed for all M3 surgeries [4, 9, 14, 17-20].

Corticosteroids are well-known adjuncts to surgery due to its powerful anti-inflammatory effects. The most commonly used forms of corticosteroids in dentoalveolar surgery include dexamethasone, which is a synthetic analog of prednisolone, and may help reducing pain, edema and trismus [14, 19, 21]. Alexander et al [21] in an extensive review of the literature observed in several studies that the benefit of corticoid use was described as “limited responses” to single doses of steroids to a significant decrease in clinical swelling, pain, and trismus. Antunes et al [14] declared that oral administration of 8 mg of dexamethasone proved effective in reducing discomfort after M3 surgeries. Vegas-Bustamante et al [19] observed that 40 mg of methylprednisolone injected into the masseter muscle in the immediate postoperative period reduces swelling, trismus and pain. Lopez Carriches et al [17] compared the analgesic efficacy of methylprednisolone versus diclofenac after surgical removal of M3 and observed none differences between groups, concluding that corticoid should not be used as routine. Grossi et al [18] evaluated the effect of submucosal administration of dexamethasone on discomfort after M3 surgery and observed limited and nonsignificant effect of corticoid on pain and trismus when compared with the control group. Despite the discussion concerning the benefit of using corticoids on M3 surgeries, it is not know the possible benefits of the concomitant use corticoid and antibiotics.

The aim of this study was to assess the efficacy of a single prophylactic dose of amoxicillin and/or dexamethasone in preventing PC after a surgical removal of a single M3 as well as evaluate its effect over trismus, edema and pain using a double-blind placebo controlled randomized clinical trial.

## Materials and Methods

This study is a single center, prospective, randomized, double blind, placebo controlled clinical trial for comparison of use of amoxicillin (AMO) and dexamethasone (DEX) combined or not for prevention of postoperative complications (alveolar osteitis (AO); and alveolar infection (AI)), pain, trismus and edema following inferior third molar surgeries. This research was approved by Ethical Committee under number 030/2009 (http://unoesc.edu.br/unoesc/pesquisa/comite-de-etica-em-pesquisa).

Four groups were included: Group 1 (G1) included a prophylactic dose of 2g of amoxicillin and 8 mg of dexamethasone; Group 2 (G2) included a prophylactic dose of 2 g of amoxicillin and 8mg of placebo; Group 3 (G3) included a prophylactic dose of 8 mg of dexamethasone and 2 g of placebo and; Group 4 (G4) included two prophylactic doses of placebo mimicking amoxicillin (2 g) and dexamethasone (8 mg). Both drugs AMO and DEX were bought form commercially available and re-packed in a compounding pharmacy to standardize the color of capsules. Both drugs and placebo were then packaged together according to the group to ensure the blinding and the random process.

As inclusion criteria patients must be considered healthy or meet the American Society of Anesthesiologists classification status I (ASA I- normal healthy patients). All patients were submitted to blood tests (complete blood count and blood glucose) to ensure the health condition. Patients with anemia (hemoglobin of <13 g/dL in males - a hematocrit (Hct) of about 39; and <12 g/dL in females - Hct about 36) or with total leucocytes count < 4,000 cells/mL or neutrophils < 2,000 cells/mL were excluded (as well as leukocytosis). Patients with glucose parameters beyond normal limits (65 to 110 g/dL) were excluded. History of allergy, recent uses of antibiotics, active pericoronitis (local infection with presence of symptom or pus) and fractured root left in the socket were also excluding criteria. Panoramic radiography was taken from all patients and the difficulty of the surgery was evaluated through Pederson Index following Pell and Gregory classification, and the Winter classification as described elsewere [22, 23]. That scoring (3 - 10 points) criteria takes into account the inclination of the longitudinal axis of the molar (Mesioangular: 1; Horizontal/Transverse: 2; Vertical: 3; and Distoangular: 4); depth, with respect to occlusal plane (Level A: 1; Level B: 2; Level C: 3); and available space, with respect to ascending mandibular ramus (Class I: 1; Class II: 2; Class III: 3). The sum of the scores may be classified as little difficulty, 3 to 4 points; moderate difficulty, 5 to 6 points and; great difficulty, 7 to 10 points. The preliminary evaluation must predict surgical flap and ostectomy (inclusion criteria).

Patients who meet all inclusion criteria were invited to participate. After careful explanation all the patients taking part in the study fully understood its scope and signed the informed-consent form. Patients were assembled in one of four groups’ trough raffle and received the medications/placebo between 1 to 1½ hours before surgery. A single inferior third molar was included for surgery. Surgeries were performed by a last year dentistry student with a standardized technique, but under direct orientation and presence of one oral and maxillofacial surgeon of the team. All surgeries were performed between 5 to 6 p.m. and the extractions were carried out under similar clinical conditions. Procedures were performed under the most rigorous control of microbiologic contaminants and included sterile surgical apron, sheets and gloves. Dental handpieces, drills and surgical instruments were sterilized in autoclave. Sterile saline solution was used for lavage of the alveolus socket and bur refrigeration. Research auxiliaries took notes of the surgery development such as time, surgical accidents, numbers of anesthetic cartridges (mepivacaine 2% with 1:100,000 epinephrine or articaine 4% with 1:100,000 epinephrine, according to the surgeon preference) and other variables of interest.

Due to ethical reasons analgesics were prescribed to all patients (acetaminophen 750 mg 4 times a day, for two days, through oral route) and non-steroidal anti-inflammatory drugs (NSAIDs- sodium diclofenac 50 mg, 3 times a day, for two days, through oral route), however was allowed to the patient to discontinue these drugs (acetaminophen and/or sodium diclofenac) or even do not take it if no symptoms were present but, patients were advised to take the analgesic tablet as soon as their pain started. To the patient was also allowed to continue with the medication after the prescribed period, however it was necessary a clinical examination and careful record of how many tables of both drugs were necessary to control pain in the whole postoperative period. Each patient received a diary to be filled daily considering pain, edema, trismus and the number of capsules taken and had to have completed and returned their postsurgery diary at the reevaluating day. The postoperative cares and recommendations were similar to all patients and were directed mainly to keep the blood clot in place, avoiding rigorous mouthwash, ice pack in the first 24 h, maintaining a sensible oral hygiene and keep at least 12 hours rest.

### Criteria for alveolar osteitis (AO) and alveolar infection (AI)

The criteria for AO and AI were based on the clinical conditions as previously described by several authors [1, 5]. When present, those conditions were checked and confirmed by at least one of the oral and maxillofacial surgeons of the research team. Patients with diagnosis of AO or AI were treated for the condition and excluded for subsequent analysis (pain and edema).

### Pain Evaluation

**Figure 1:**
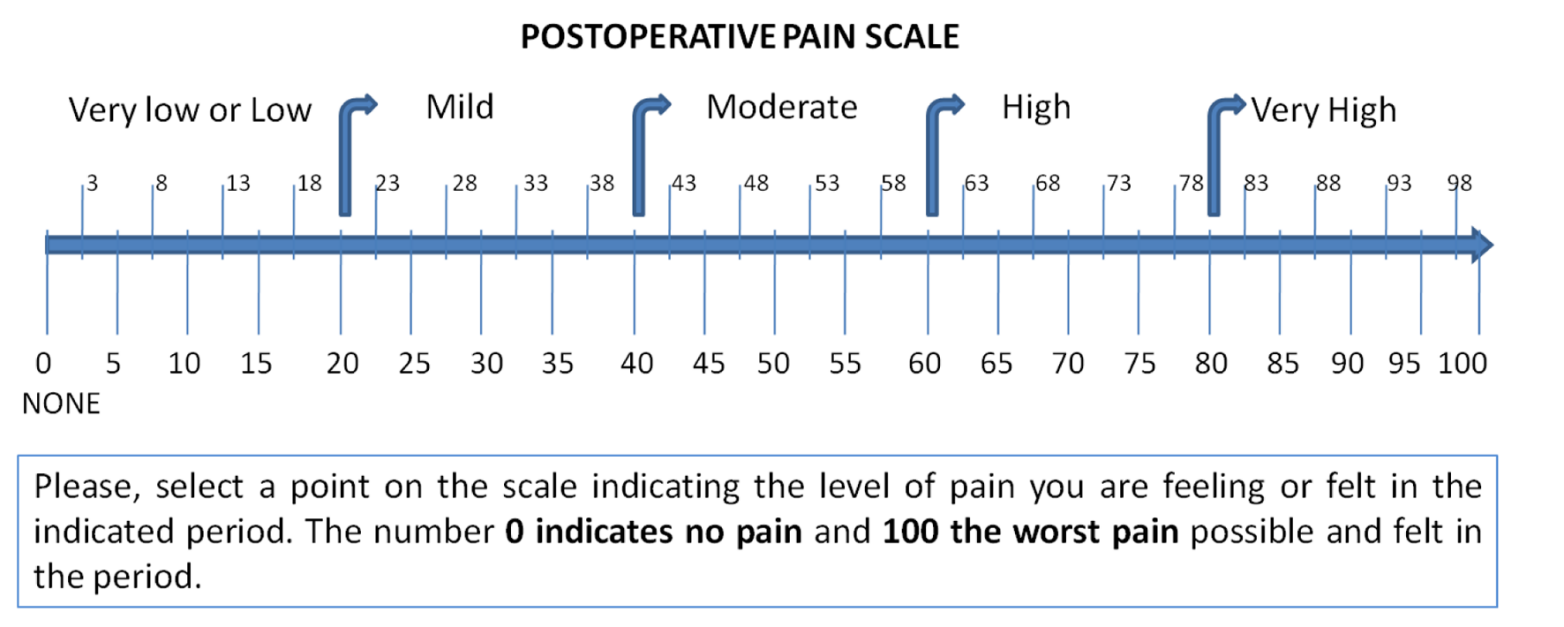
Visual analogue scale used in patients diary for guide in self-score pain.

Pain evaluation was self-rated through a visual analogue scale (0 - 100) (Fig. 1), 10 times in the course of 5 days, starting at 5 and 6 hours after surgery, at waking time and at the end of the day (standardized between 6 to 8 p.m.) for days 1 to 3 and end of the day for days 4 and 5. Patient must record pain considering the highest experience in the period between the previous annotation. Since the development of PCs dramatically increases the postoperative pain [13], cases diagnosed as postoperative complication (PC) were excluded of the pain analysis.

### Edema evaluation

Postsurgical facial edema is difficult to quantify accurately because it involves 3 dimensions of measurement. Due to this characteristic the evaluation for edema was based in patient experience (self-rated) through a visual analogue scale (0 - 100). Edema was evaluated five times always at the end of the day and starting at the end of the first postoperative day. Cases diagnosed as postoperative complication (PC) were excluded from edema analysis due to the fact that the edema which usually maximizes or continues to expand after 3 days is thought to be additional swelling resulting from infection [21].

### Trismus evaluation

Trismus was evaluated according to its presence or absence based in clinical observation and patient report of having any significant limitation of mouth opening (half of normal mouth opening).

### Data analysis

The data collected were analyzed as appropriate in statistically software (SPSS statistical software, version 20.0; SPSS^®^ Inc., Chicago, IL) in a level of significance of P ≤ 0.05.

## Results

Fifty patients met the inclusion criteria and were randomly distributed in one of the four groups. Groups 1, 2 and 4 received 12 patients each (24%) and G3 receive 14 (28%). The homogeneity of the groups was assured by a series of analysis described in Table 1, with no differences observed between groups related to several variables of interest. It was observed one case of AI (2%) and two of AO (4%) resulting in three PC (6%). Thirteen patients (26%) report significant limitation of mouth opening. No statistical differences were observed for PC and trismus related to therapeutic groups and the distribution of both variables according to groups can be seen in Table 1. Even if added G1 + G2 considering the use of antibiotics or not (G3 + G4), the results did not showed significant differences for preventions of PC (Fisher’s Exact Test, P = 1.0 (PC with antibiotics 1 yes/23 no; PC without antibiotics 2 yes/24 no)). The use of antibiotics showed an absolute risk reduction for (ARR) PC development of 3.52% and the number needed to treat (NNT) or, the number of patients need to treat to prevent one additional bad outcome is 29. None late PC development was observed in follow-up. However, for trismus development, antibiotics showed an ARR of 17.95% and a NNT of 6.

**Table 1:** Analysis, Distribution and Homogeneity of Variables of Interest in Therapeutic Groups and Results for Postoperative Complication (Alveolar Osteitis + Alveolar Infection) and Trismus (n:50)

Variables		G1 (n:12)	G2 (n:12)	G3 (n:14)	G4 (n:12)	Statistical Test	P value
Sex	Male	2	6	3	2	Pearson Chi-Square	0.1
	Female	10	6	11	10		
Age (Mean)		23.2	22.8	21.5	22.5	Kruskal Wallis	0.7
Teeth sectioning	Yes	6	8	4	5	Pearson Chi-Square	0.2
	No	6	4	10	7		
Pell and Gregory and Winter classification scores (Mean)		5.7	6.5	6	5.8	Kruskal Wallis	0.1
Difficulty Rating	Little difficulty	1	-	1	-	Pearson Chi-Square	0.3
	Moderate difficulty	9	5	7	9		
	Great difficulty	2	7	6	3		
Smoker	Yes	1	0	1	0	Pearson Chi-Square	0.6
	No	11	12	13	12		
Regular alcohol consumption	Yes	4	3	1	0	Pearson Chi-Square	0.08
	No	8	9	13	12		
Oral hygiene	Good	11	12	11	9	Pearson Chi-Square	0.2
	Not good	1	0	3	3		
Sodium Diclofenac (Mean)	Tablets	6.2	4.6	4.8	5.4	Kruskal Wallis	0.5
Acetaminophen (Mean)	Tablets	7.5	9.1	8.2	8.7	Kruskal Wallis	0.9
Ice use (Mean- minutes)		57.9	44.1	52.5	56.6	Kruskal Wallis	0.6
Surgical time (Mean- minutes)		51	56.2	47.3	51.2	ANOVA	0.8

**Results**							

Postoperative Complication (alveolar osteitis + alveolar infection)	Yes	0	1(AO)	1(AI)	1(AO)	Pearson Chi-Square	0.7
Trismus	Yes	1	3	4	5	Pearson Chi-Square	0.3
	No	11	9	10	7		

AO: Alveolar Osteitis; AI: Alveolar Infection; G1: Therapeutic Group 1 (amoxicillin 2 g + dexamethasone 8 mg); G2: Therapeutic Group 2 (amoxicillin 2 g + placebo 8 mg); G3: Therapeutic Group 3 (placebo 2 g + dexamethasone 8 mg); G4: Therapeutic Group 4 (placebo 2 g + placebo 8 mg).

**Figure 2:**
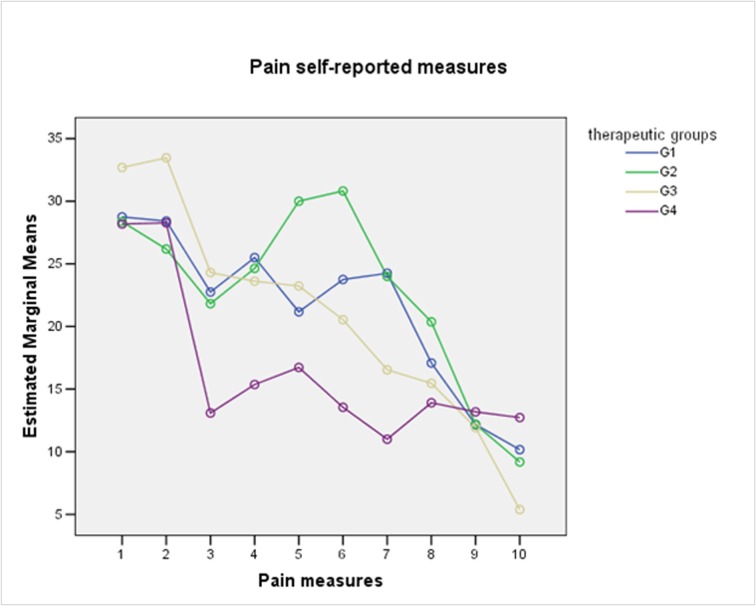
Self-reported measures for pain according to therapeutic groups.

**Figure 3:**
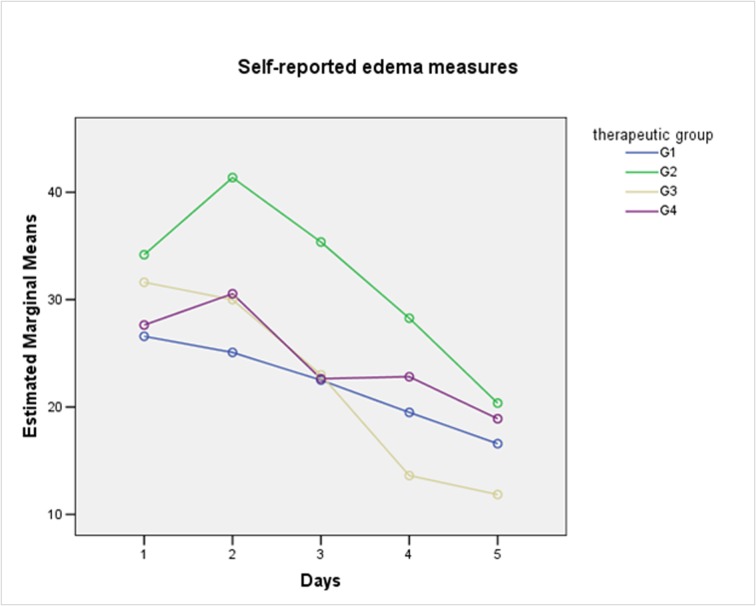
Self-reported measures for edema according to therapeutic groups.

For pain and edema analysis, were excluded those patients with PC. Pain did not show significant differences between groups (One-Way Repeated-Measures ANOVA with Lower-bound correction, P = 0.32) (Fig. 2). From the patient perspective, edema also did not show significant differences between groups. (One-Way Repeated-Measures ANOVA with Lower-bound correction, P = 0.41) (Fig. 3). The combined use of amoxicillin and dexamethasone did not show a statistical significant difference in improvement of self report scores for edema (G1 against G2 + G3 + G4) (One-Way Repeated-Measures ANOVA with Lower-bound correction, P = 0.39); or for pain (One-Way Repeated-Measures ANOVA with Lower-bound correction, P = 0.48).

The Pell and Gregory and Winter classification scores (3 - 10) showed a positive and weak to moderate correlation with pain scores from the beginning of the first day postoperative to the end of the fourth day (Spearman’s rho Correlation -1-tailed: First day-end: r_s_ 0.27, P = 0.03; Second day-beginning: r_s_ 0.43, P = 0.001; Second day-end: r_s_ 0.35, P = 0.007; Third day-beginning: r_s_ 0.36, P = 0.006; Third day-end: r_s_ 0.41, P = 0.002; Fourth day-beginning: r_s_ 0.27, P = 0.03; Fourth day-end: r_s_ 0.27, P = 0.03). None correlation between Pell and Gregory and Winter classification scores with self reported edema scores, however edema showed to be positive and weakly correlated with age but just for days 2 and 3 postoperative (Spearman’s rho Correlation -1-tailed: First day: r_s_ 0.32, P = 0.014; Second day: r_s_ 0.29, P = 0.024).

## Discussion

The use of antibiotics may bring some undesirable effects such biological resistance, allergic and gastrointestinal reactions and increase of costs. Decades of overuse and misuse of antibiotics have lead to an increasing incidence of methicillin-resistant *Staphylococcus aureus* (MRSA), once a hospital acquired infection, but nowadays has been detected in increasing number in facial abscesses [24]. The aim of antibiotic prophylaxis in surgery is to prevent the possible appearance of infection in the surgical wound however, this practice for M3 surgery has been an issue of controversy. The conflicting conclusions from randomized controlled clinical trials have caused confusion in clinical practice, with advocates and opponents of antibiotic prophylaxis [3, 25]. In a Spain consensus document [26], the authors assume that the literature is contradictory for indications of antibiotic prophylaxis in oral surgery and in general terms antibiotic therapy pre- and post-operatively is recommended in those cases that have a high risk of infection or obvious clinical signs of infection. Those authors [26] establish that the criterion for choosing antibiotic prophylaxis or not must be based on the benefit and the cost of the risk.

Bezerra et al [11] compared amoxicillin versus placebo for M3 surgeries and although 50% of those patients presented inflammatory/infectious events the incidence did not differ significantly between the experimental and control groups. Those authors [11] also observed no significant difference in the level of edema between groups and concerning pain a significant difference was observed between groups only on day 7 of evaluation. According to Lacasa et al [27] for M3 surgeries there was a trend of higher infection rates when compared placebo (16%; 12/75) vesus prophylaxis (5.3%; 4/75; two active amoxicillin/ clavulanate 1000/62.5 mg tablets in a single dose before surgery), however not significant. Those authors [27] also report that the impact of antibiotic exposure on the rate of infection depending on the difficulty of the surgical procedure revealed that the antibiotic strategies were significantly more effective than placebo only among those patients subjected to ostectomy. In the present study all patients suffered ostectomy and besides there is also a trend of higher infection rates for placebo group it did not reach statistical significance.

Also in agreement with the present study Pasupathy and Alexander [12] also found none advantage in the routine use of prophylactic antibiotics for M3 surgeries. Ataoglu et al [16] also found no significant difference between the three groups evaluated and concluded that routine antibiotic prophylaxis is unwarranted for routine operations to remove third molars in healthy patients. Similarly to what was found in the present study Siddiqi et al [10] in a prospective, randomized, double blind, placebo-controlled clinical trial observed that prophylactic antibiotics did not have significant impact in the infection rate, pain, swelling and trismus for M3 surgeries. Monaco et al [28] performed a randomized clinical trial with 59 patients to evaluate the influence of antibiotic prophylaxis (2 g amoxicillin) on postoperative complications after M3 removal. Those authors [28] claim that they found a statistically significant (P = 0.01) difference between patients receiving preoperative amoxicillin and the control group in the incidence of wound infection, since for control group 1 patient developed wound infection and 31 does not (total 32), while in the control group 4 patients developed wound infection and 23 does not (total 27). We observed a very serious flaw in the test interpretation since the correct p value is P = 0.1 (Fisher exact test, Yates correction for two categories of data, one degree of freedom P = 0.2) and it is absolutely not significant. An errata for that manuscript is not yet available [28] besides the attempt to publish a “letter to editor” and being available, the manuscript have been cited [10], reinforcing the confusion. We are aware that the limitation of our study is the small sample size, however it is a highly controlled study and brings additional information to the discussion about the necessity to use or not antibiotics for M3 surgeries.

Pain, trismus, and facial swelling after the surgical removal of a third molar tooth are routine sequel due to inflammation as a result of surgery. Similarly to what was found in the present study, Kang at al [29] also observed that corticoid (20 mg of prednisolone) used as a single preoperative oral administration had no significant effect on postoperative symptoms of pain, facial edema and trismus in M3 surgeries. Besides the differences between methodological approaches, both studies evaluated the degree of postoperative symptoms using a self-reported VAS scores including pain and facial edema. Besides Antunes et al [14] declared that oral administration of 8 mg of dexamethasone proved effective in reducing pain, edema, and trismus after M3 surgeries, their results showed significant improvement only in the second day of evaluation for trismus and no differences for edema into two measures (EC-GA and Tr-OC). Those differences between studies may lie on the analysis method, due to while 5 mm of facial edema may be mathematically significant, but it may not be clinically significant and the present study tried to search the impact over the clinical self-perception. Differently to what was found by Antunes et al [14], our results suggest that single dose preoperative oral of 8 mg of dexamethasone has no significant effect on postoperative symptoms in M3 surgery.

Besides the differences between studies, Kim et al [30] observed a statistically significant relationship with one day postoperative edema with age, the degree of impaction and the duration of operation time, the present study also observed a positive correlation age related edema but for day 2 and 3 postoperative.

In conclusion, this study showed that the use of prophylactic antibiotics associated or not with prophylactic use of corticoid in a single dose regimen did not have a significant effect on postoperative complications (alveolar osteitis and alveolar infection) and did not have a significant improvement of postoperative discomfort such as pain, edema and trismus for third molar surgery. The authors recommend that antibiotic and corticoid prophylaxis should not be administered routinely in single dose when third molars are removed in healthy and young patients since it did not produce any clear benefit.
